# Role of Electrostatic Interactions on Supramolecular Organization in Calf-Thymus DNA Solutions under Flow

**DOI:** 10.3390/polym10111204

**Published:** 2018-10-28

**Authors:** L. Mónica Bravo-Anaya, Denis C. D. Roux, J. Félix Armando Soltero Martínez, Francisco Carvajal Ramos, Frédéric Pignon, Oonagh Mannix, Marguerite Rinaudo

**Affiliations:** 1 University Grenoble Alpes, CNRS, Grenoble INP, LRP, F-38000 Grenoble, France; monik_ayanami@hotmail.com (L.M.B.-A.); frederic.pignon@univ-grenoble-alpes.fr (F.P.); 2 Departamento de Ingeniería Química, Universidad de Guadalajara, Blvd. M. García Barragán #1451, Guadalajara C.P. 44430, México; jfasm@hotmail.com; 3 CUTonalá, Departamento de Ingenierías, Universidad de Guadalajara, Nuevo Periférico #555 Ejido San José Tatepozco, Tonalá C.P. 45425, México; iq_fcr@yahoo.com.mx; 4 European Synchrotron Radiation Facility, 38000 Grenoble, France; oonmannix@gmail.com; 5 Biomaterials Applications, 6 Rue Lesdiguières, 38000 Grenoble, France

**Keywords:** DNA, external ionic concentration, rheology, birefringence, SAXS, electrostatic interactions

## Abstract

Previous investigations were conducted on two concentrations of DNA solution: 4 mg/mL, for which it has been shown that no supramolecular organization is induced under flow at low shear rates; and 10 mg/mL, in which a liquid crystalline-type texture is formed under flow at low shear rates, attesting to an orientation of pre-organized chains. Rheological experiments are discussed and their results supported by small-angle X-ray scattering (SAXS) and flow birefringence visualization experiments. Scattering from polyelectrolytes has a characteristic signal, which is here observed in SAXS, showing a strong correlation peak between charged chains in water, for both concentrations. This peak is weaker in the presence of 0.01 M NaCl and suppressed in salt excess at 0.1 M NaCl. No plateau in the *σ*($\dot{\gamma }$) plot was observed in analysis of rheological experiments on low DNA concentration (4 mg/mL). As typically observed in polyelectrolyte systems both the dynamic moduli and shear viscosity were higher in water as electrostatic forces dominate, than in the presence of salt, especially at low shear rates. The rheological results for concentrations of 0.01 M NaCl are lower than in water as expected due to partial screening of electrostatic repulsions. Rheological data for concentrations of 0.1 M NaCl are unexpected. Electrostatic forces are partially screened in the low salt concentration, leading to a drop in the rheological values. For high salt concentration there are no longer interchain repulsions and so steric interactions dominate within the entangled network leading to the subsequent increase in rheological parameters. Regardless of the solvent, at high shear rates the solutions are birefringent. In the 10 mg/mL case, under flow, textures are formed at relatively low shear rate before all the chains align going to a pseudonematic liquid crystalline phase at high shear rate. The electrostatic repulsion between semi-rigid chains induces a correlation between the chains leading to an electrostatic pseudo-gel in water and loosely in 0.01 M NaCl at low stress applied. To the best of our knowledge, this is the first time that such behavior is observed. In 0.1 M NaCl, DNA behavior resembles the corresponding neutral polymer as expected for polyelectrolyte in salt excess, exhibiting a yield stress. When texture appears in water and in 0.01 M NaCl, a critical transition is observed in rheological curves, where the viscosity decreases sharply at a given critical shear stress corresponding to a plateau in the *σ*($\dot{\gamma }$) plot also observed in creep transient experiment.

## 1. Introduction

DNA is a semi-rigid polyelectrolyte with a high persistence length (Lp~50 nm) [1,2,3]. Understanding electrostatic interactions between stiff chain segments in DNA and polyelectrolyte solutions is important, both fundamentally and for biological applications [4]. These interactions depend on the charge density of the polyelectrolyte favoring parallel alignment of the chains, and on the external salt concentration screening the electrostatic repulsions [5]. In addition, DNA may be considered a good model for the study of dynamics and macroscopic properties of semi-rigid macromolecules in solution.

Due to condensed counterions in 1-1 electrolytes, there exists a range of distances less than the Debye length where polyions attract each other [6,7,8,9,10]. Birefringence has previously shown that textures corresponding to orientated, ordered domains are induced under shear flow for DNA concentrations higher than 5 mg/mL at low ionic concentration [11]. A schematic representation of the evolution of these solutions at rest and under flow was proposed [11]. Understanding the nonlinear behavior of entangled DNA solutions using rheology has been the subject of previous work. Of particular interest is the transformation from steady-state flow to so-called bulk shear inhomogeneities.

From a mechanical point of view, in the steady-state flow curve, the onset of a stress plateau above a critical shear has been associated with the existence of nonhomogeneous flow [11]. In that work, the supramolecular organization in entangled calf-thymus DNA solutions under flow was studied in a large DNA concentration range between 2 and 10 mg/mL in TE buffer (ionic concentration equivalent to 0.01 M) at pH = 7.3 and at 20 °C. The birefringence observed at these conditions was directly related to the rheological behavior of DNA.

To complete our previous work, the rheological behavior of DNA in aqueous solution is studied: at two polymer concentrations (4 and 10 mg/mL); in water, and in the presence of two ionic concentrations, 0.01 and 0.1 M NaCl, to investigate the influence of ionic concentration on rheological behavior and on birefringence induced under flow. The polymer concentrations are chosen such that DNA is in the semidilute regime with a low degree of entanglement. SAXS experiments complete the data studying in the same solvent conditions, long-range interactions between chains.

The aim of this paper is to identify the appearance of different flow regions and, particularly, the conditions at which the onset of a stress plateau appears in flow experiments in relation to the electrostatic repulsions. Rheometric measurements in combination with flow birefringence observations are proposed to reach a better understanding of the mechanisms involved in this particular heterogeneous rheological behavior and the existence of a plateau in the shear stress/shear rate flow curve.

## 2. Materials and Methods

### 2.1. Materials and Solutions Preparation

High-molecular weight calf-thymus DNA (*M*_W_ = 6,560,000 g/mol) was used to prepare solutions at concentrations of 4 and 10 mg/mL [11]. Ultrapure water and NaCl anhydrous were used to obtain different external ionic concentrations: 0, 0.01, and 0.1 M NaCl. The double helix conformation of DNA is stabilized under these conditions. Vials were closed and sealed with Parafilm^®^ to prevent water evaporation and concentration changes. Solutions were stored at 4 °C to prevent degradation. Sigma-Aldrich Company (St. Louis, MO, USA) supplied all reagents.

### 2.2. Rheometric Measurements

DNA rheology was studied at 25 °C using a DHR-3 rheometer from TA Instruments Company (New Castle, DE, USA) and a MCR-501 rheometer from Anton-Paar Company (Graz, Austria). Two geometries were used: a steel cone-plate geometry with 40 mm diameter and an angle of 0.035 rad was used in DHR3 rheometer and a glass plate–plate geometry with a 50 mm diameter was employed with the MCR501 rheometer to observe birefringence under crossed polarizers. Birefringence visualizations were integrated through the gap with a wave vector in the direction of the velocity gradient. A solvent cap was used to prevent solvent evaporation.

To define the linear viscoelastic regime oscillatory strain sweeps were undertaken at an angular frequency of 10 rad/s in the strain range from 0.01% to 100 %. Frequency sweeps were performed at 1% strain within the linear viscoelastic regime of *G’* and *G’’* in a frequency range *ω* from 0.01 to 100 rad/s, with at least 5 points per decade. Simple steady state shear was measured at shear rates from 10^−3^ to 1 000 s^−1^ with at least 5 points per decade. Due to thixotropic behavior of 10 mg/mL DNA solution, in order to reduce the time to obtain the steady state, increasing/decreasing ramps were imposed. Firstly, a rapid shear rate increase from 0 to 100 s^−1^ was applied in 160 s followed directly by a decreasing ramp in 3000 s. 

Transitory experiments in creep flow consist of applying constant stresses from 10^−2^ to 20 Pa and observing the creep flow over a time up to 10^3^ s for each applied stress. In the first part of the shear rate (time response), the transient regime is characterized by damped oscillations due to a coupling between tool inertia and solution viscoelasticity. Final constant shear rate corresponds to the steady state.

### 2.3. Flow Birefringence Visualizations

**Figure 1:**
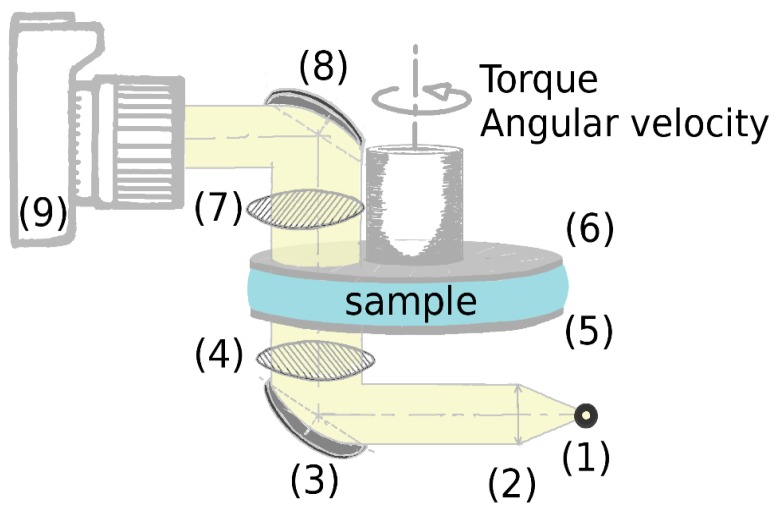
Schematic description of the home-made birefringence set-up on the rheometer MCR-501. (1) White source, (2) lens, (3) and (8) mirrors, (4) and (7) crossed polarizers, (5) and (6) plate–plate geometry, and (9) camera.

Birefringence was tested using a glass plate–plate configuration with an adapted gap, of at least 0.2 mm, such that the geometry was filled completely with DNA solution. This geometry is placed between crossed polarizers, one below and another just above the sample (Figure 1). The sample was illuminated vertically by a white source of light reflected by a mirror placed at 45° below the first polarizer under the geometry. A second mirror, placed at 45° above the upper polarizer, collected pictures at different imposed shear rates with CMOS device cameras (Canon 100 D, Canon, Tokyo, Japan or Sony DSLR-A580, Sony, Minato, Japan) and adapted commercial zooms.

### 2.4. Small-Angle X-Ray Scattering (SAXS) Measurements

The SAXS experiments were performed on ID02 high brilliance beamline at European Synchrotron Radiation Facility (ESRF, Grenoble, France). DNA solutions were analyzed under controlled temperature (25 ± 1 °C) in a flow-through capillary cell (diameter ~2 mm). An incident monochromatized X-ray beam at wavelength of 0.995 Å was used. Scattered intensities were recorded on a two-dimensional CCD detector at sample-to-detector distances of 0.75 and 8 m. The scattering intensity distribution as a function of scattering vector was obtained by radial integration of 2D scattering pattern. Background subtraction was done using a cell filled with water for all samples. This is not a perfect background for solutions with high salt concentration, as seen in the increased intensity in the high-q region. As scattering from the salt ions makes no contribution in the low-q region this does not change outcome of the analysis.

## 3. Results and Discussion

### 3.1. X-Ray Scattering

Both X-ray scattering and rheological experiments were performed in identical experimental conditions. The influence of ionic concentration is examined by adding external salt. A correlation peak is observed in the SAXS data, this is evidence of long-range electrostatic repulsions. These repulsions are caused by interactions between polymer chains forming a local network with a preferential interchain distance.

**Figure 2:**
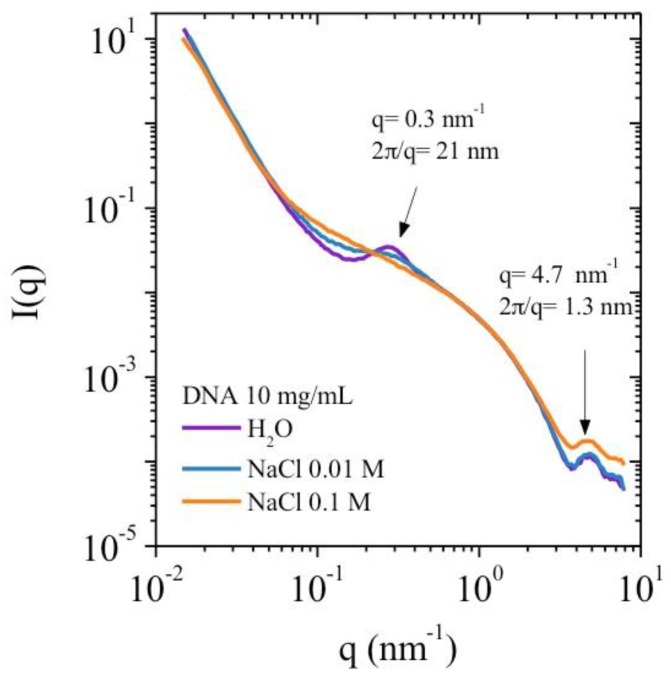
Small-angle X-ray scattering (SAXS) intensity *I* (*q*) as a function of the wave vector *q* at different external salt concentrations. DNA solution at 10 mg/mL at 25 °C.

The correlation peak for polyelectrolyte solution in absence of external salt is located at q* such as 2 π/q* = 21 nm at 10 mg/mL DNA solution in water. It is progressively depressed in the presence of external salt as shown for 0.01 M NaCl and is suppressed in 0.1 M NaCl. Correlation peak extinction in salt excess is typically observed in polyelectrolyte solutions and also observed in neutron and light scattering experiments [12,13,14,15]. At high *q* values (*q* > 3 nm^−1^), the form factor of DNA is independent of the salt content (Figure 2) and agrees with the model given by Pringle and Schmidt [16].

**Figure 3:**
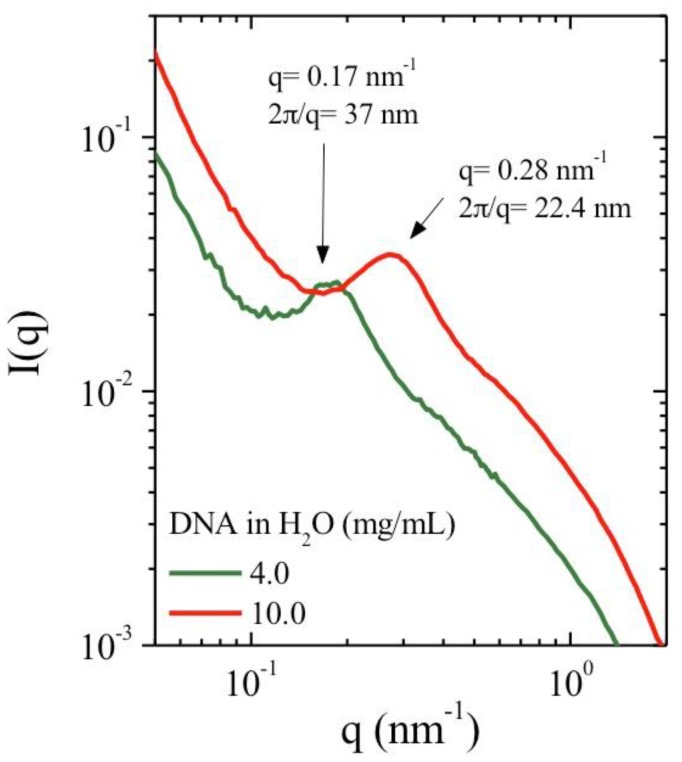
SAXS intensity *I* (*q*) as a function of the wave vector *q* for 4 and 10 mg/mL DNA concentrations at 25 °C.

The influence of polyelectrolyte concentration is shown in Figure 3. The SAXS intensity profile is presented in the absence of external salt at 4 and 10 mg/mL q*, corresponding to the maximum in the SAXS intensity as a function of *q*, decreases when the polymer concentration (*C*_p_) decreases as found in the literature. The values of q* vary following a scaling law q*~*C*_p_^1/2^ in agreement with a hexagonal packing with a correlation distance between packed strands, indicating a preferential distance between chains. At 4 mg/mL q* = 0.17 nm^−1^, which gives a predicted value of q* = 0.27 nm^−1^ at 10 mg/mL using this scaling law. This value is in agreement with the experimentally determined value of q* = 0.28 nm^−1^.

These results confirm our hypothesis on the formation of organized chains [11]. The SAXS values obtained indicate a nearly homogeneous distribution of stiff interacting chains throughout the solution. This agrees with the schematic representation given by Odijk for the case where polymer persistence length is greater than the distance between two chains [4].

To reflect the role of interchain electrostatic repulsion the DNA Debye lengths were estimated. This includes the DNA counterion contribution (*C*_p_ (ion/L) = [(0.12/370) × *C* (g/L)] where 0.12 is the activity coefficient of counterions and 370 the average molar mass of the nucleic repeat unit; and the ionic contribution of external salt (Table 1). In 0.1 M NaCl the behavior should approach the corresponding neutral polymer. In these experimental conditions, the influence of the ionic concentration, as demonstrated by the estimated Debye length, is low compared to the intrinsic value of the persistence length, around 50 nm.

Finally, it is important to mention the large increase of *I* (*q*) at low *q* values indicating the formation of supramolecular structure as proposed in our model [11]. Moreover, the intensity profile at low *q* values (*q* ~ 10^−2^ nm^−1^) agrees with the existence of aggregates with constant dimension for H_2_O and 0.01 M NaCl [17]. In salt excess condition, *I* (*q*) slope is lower indicating a significant change of structure **(Figure 2**). Unfortunately, this does not allow direct determination of the persistence length from experimental data as obtained previously on another polyelectrolyte [18].

### 3.2. Birefringence and Texture under Shear

Data previously obtained for DNA solution at 10 mg/mL, in TE buffer at ionic concentration around 0.01 M, were completed with birefringence observations, as a function of shear rate. The two polymer concentrations were chosen; as at 4 mg/mL the absence of birefringent texture was previously demonstrated, while texture was observed at 10 mg/mL. This enables investigation of the influence of texture on rheological behavior.

**Figure 4:**
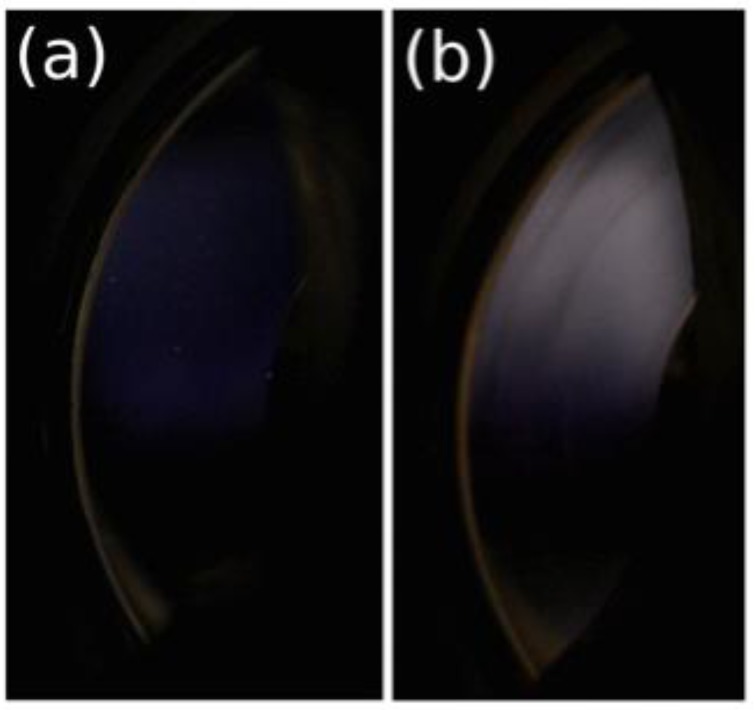
Birefringence of DNA solution at 4 mg/mL in 0.1 M NaCl under shear (**a**) at 10^−3^ s^−1^ and (**b**) at 500 s^−1^.

**Figure 5:**
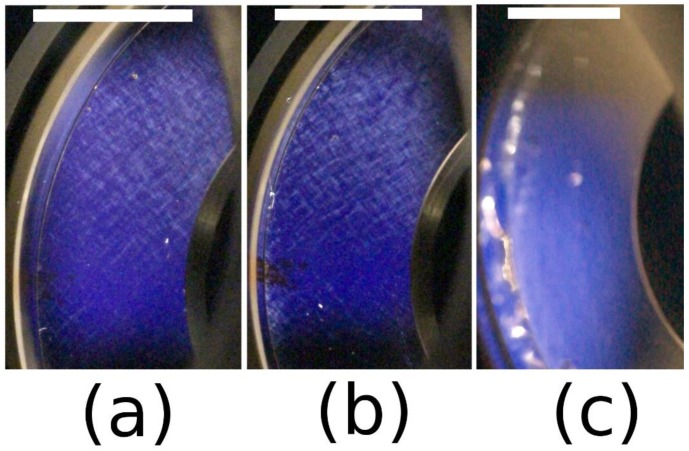
Supramolecular organization of 10 mg/mL DNA solution sheared at 25 °C: (**a**) Texture obtained in water; (**b**) in 0.01 M NaCl; and (**c**) heterogeneous birefringence in 0.1 M NaCl. The white bars represent the scale of 10 mm. Two videos for solutions in water and in 0.1 M NaCl under shear are joined in SI.

In Table 2 it is shown that salt has a critical role in observing birefringence and birefringent textures. Figure 4 and Figure 5 present examples of birefringence and the textures observed.

These data show no texture is formed at concentrations of 4 mg/mL, confirming our previous observations [11], however birefringence is observed at relatively high shear stress (~4.5 Pa). Our model of DNA consists of the presence of ordered domains, as evidenced by the correlation peak in SAXS data. These domains are loosely connected by extended single chains. At high shear stress the domains are fully orientated, forming a pseudonematic phase. In a previous study, the orientation of semi-rigid chains was directly demonstrated by neutron scattering on xanthan (a semi-rigid polysaccharide) solution under shear 15.

The role of polymer concentration on the supramolecular organization of DNA in solution is demonstrated for the 10 mg/mL DNA concentration. Birefringence occurs at low shear rates, but larger shear stress due to the high solution viscosity. At 10 mg/mL, liquid crystalline-like textures occur over shear rates of 1 s^−1^ confirming our previously published data [11]. In the presence of salt at 0.1 M NaCl, heterogeneous birefringent elongated domains appear under shear. The density of these domains progressively increases to form an almost homogeneous nematic phase. In all these experiments, a slightly negative normal force is observed when chains are orientated. It increases when the textures appear and become slightly positive.

### 3.3. Rheological Behavior

To complete our previous rheological experiments on DNA solutions [11], we examined the rheological behavior at different external salt contents: in the absence of salt, and at 0.01 and 0.1 M NaCl. Electrostatic repulsions are maximum in the absence of salt and progressively screened with the addition of salt. At 0.1 M, in which the Debye length is small (Table 1), the behavior goes to that of the corresponding neutral polymer involving steric hindrance between semi-rigid chains. The influence of external salt on viscometric measurements is well described in the literature [5]. Two different polymer concentrations are tested to evidence clearly the influence of texture on the rheological behavior in the semidilute regime, 4 mg/mL being the model for absence of texture.

#### 3.3.1. Behavior at 4 mg/mL

This solution is considered as a model for an entangled semidilute system in which the role of salt concentration, intrinsic stiffness, and absence of “liquid crystalline” ordered domains or texture are investigated as a function of the ionic concentration.

##### Dynamic Measurements

**Figure 6:**
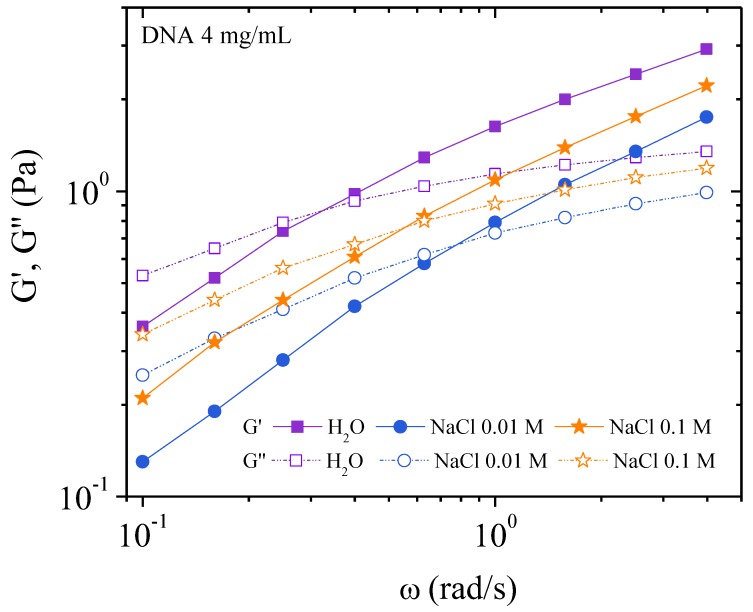
Elastic (*G*’) and viscous (*G*”) moduli as a function of the angular frequency at 25 °C for different solvent ionic concentrations 0, 0.01 M, and 0.1 M NaCl for DNA 4 mg/mL.

Rheological experiments were performed in the linear domain. The influence of external salt concentration (*C*_s_) is shown in Figure 6 and summarized in Table 3. It is seen that the bulk relaxation time is longer in water, as is the stiffness of the system, due to stronger interchain repulsion requiring a stronger stress to perturb the supramolecular structure composed of correlated chains.

The *G*’-*G*’’ crossing transition *ω*_0_ does not increase linearly with salt concentration. Firstly, *ω*_0_ increases upon addition of salt (0.01 M NaCl). This demonstrates a decrease in electrostatic forces, further evidenced by the reduction of the correlation peak in SAXS. Then ω_0_ decreases upon further addition of salt (0.1 M NaCl). This state resembles that of the corresponding uncharged polymer with stronger entangled contacts than in the case of simple electrostatic repulsion (seen for the 0.01 M NaCl concentration) leading to an increase in *G*’.

##### Flow Measurements

**Figure 7:**
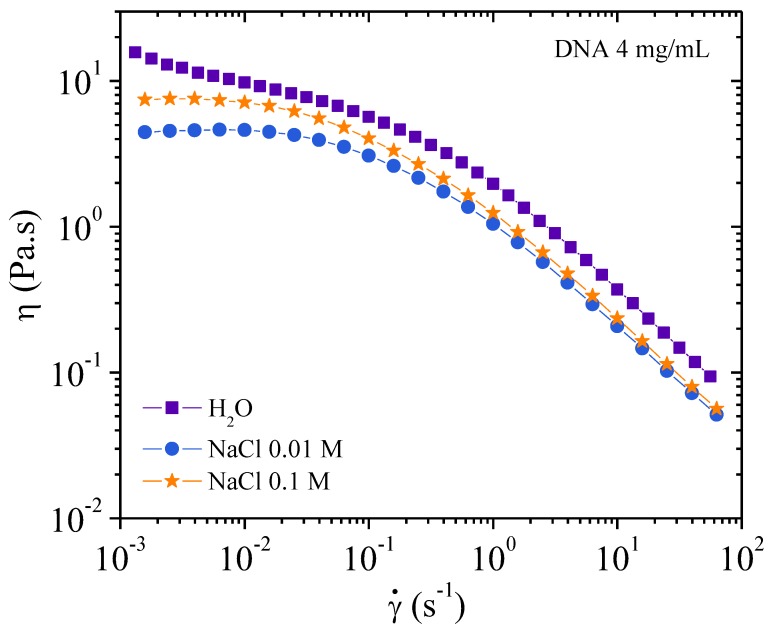
Flow viscosities of 4 mg/mL DNA solutions as a function of the shear rate at 25 °C solvents with different ionic concentrations: 0, 0.01, and 0.1 M NaCl.

Viscosities as a function of the shear rate are plotted in Figure 7. The well-known behavior of polyelectrolyte viscosity is observed in water. In absence of external electrolyte, the viscosity increases rapidly when the shear rate decreases due to strong electroviscous effects [19,20]. In 0.01 M NaCl, the screening gives a pseudo plateau at low shear rate when the ionic contribution of DNA is in the same range as the solvent ionic concentration as discussed previously [5]. 

In excess of salt at 0.1 M NaCl, the DNA solution behaves as the corresponding neutral polymer with a viscous plateau below 0.02 s^−1^. The viscosity is lower than DNA solution in water but larger than in 0.01 M NaCl due to screening of electrostatic long-range interactions. This is directly related to the dynamic behavior discussed previously (Figure 6). Over $\overset{´}{\gamma }$ > 1 s^−1^, the viscosities are nearly the same in both NaCl-containing solvents corresponding to progressive flow alignment. Birefringence measurements show a progressive increase interpreted as an alignment of domains culminating in the alignment of all chains forming a pseudonematic phase (see pictures in Figure 4). To demonstrate the validity of the dynamic and shear viscosities, the Cox–Merz representation is given in Figure 7.

**Figure 8:**
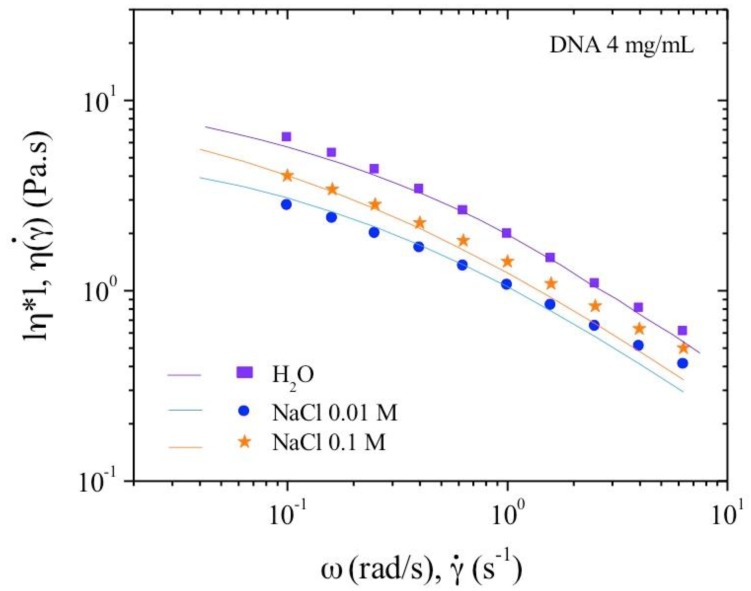
Cox–Merz overlay for 4 mg/mL DNA solution at 25 °C in different ionic concentrations: 0, 0.01, and 0.1 M NaCl. Continuous lines represent the flow viscosities and the dots represent dynamic oscillatory viscosity measurements.

Figure 8 shows the complex viscosity derived from dynamic experiments and viscosity from flow behavior are in agreement, for the frequency range covered here, corresponding to the Cox–Merz rule. In the presence of external salt, there is dissociation of the two types of viscosity curve over 1 s^−1^, with flow viscosities being lower than the complex viscosities. This is related to the screening of electrostatic repulsions and subsequent increase in the elastic contribution of the chains. 

Observing a decrease in viscosity in polyelectrolyte solutions is a normal behavior upon increasing salt concentration due to a decrease in the electroviscous effects. However, upon complete electrostatic screening, as in the case of 0.1 M NaCl, the viscosity increases at low shear rate compared with DNA solution in 0.01 M NaCl. This is due to the large steric interactions present in the semidilute entangled solution.

**Figure 9:**
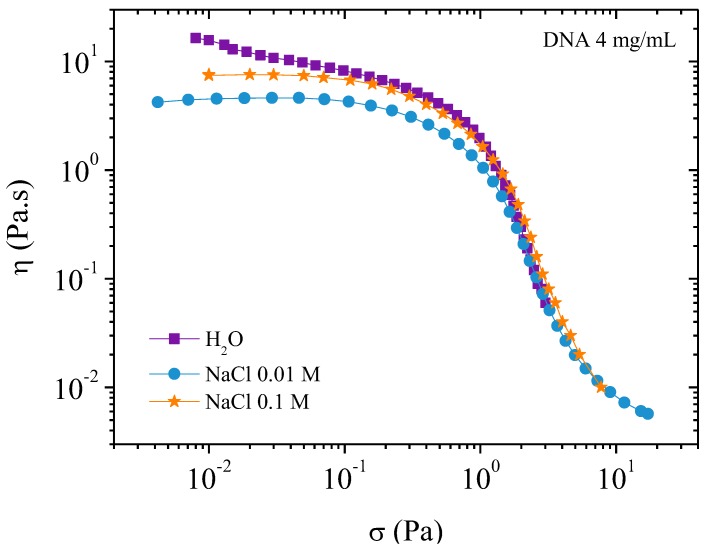
Viscosity as a function of the applied shear stress on 4 mg/mL DNA solutions in different solvents: 0, 0.01, and 0.1 M NaCl at *T* = 25 °C.

In Figure 9, the viscosity is plotted as a function of the applied shear stress. At low shear stresses the viscosity of the 0.1 M NaCl solution is lower than that of water but higher than the 0.01 M NaCl solution. A plateau is observed when NaCl is present but not in water alone. In water the viscosity increases rapidly at very low shear stress this is related to the existence of a yield stress caused by an electrostatic network formation. This point will be discussed later for higher polymer concentration.

Progressively increasing the stress induces orientation of the chains regardless of the ionic concentration. This leads to the curves superimposing for stresses around 2 Pa (over 1 s^−1^) corresponding to the appearance of birefringence, and for stress greater than 4 Pa complete orientation of chains and a pseudonematic phase is observed. Figure 9 shows a smooth transition in the *η* ($\overset{´}{\gamma }$) curve confirming the absence of a plateau on $\sigma \left(\dot{\gamma }\right)$ representation as shown in our previous work.

#### 3.3.2. Behavior at 10 mg/ML

Under the same conditions as for DNA 4 mg/mL solution, dynamic experiments were performed in the linear domain. The important difference concerns the lower stress at which birefringence appears, and the presence of texture in water and low ionic concentration (Table 2, Figure 5).

##### Dynamic Measurements

**Figure 10:**
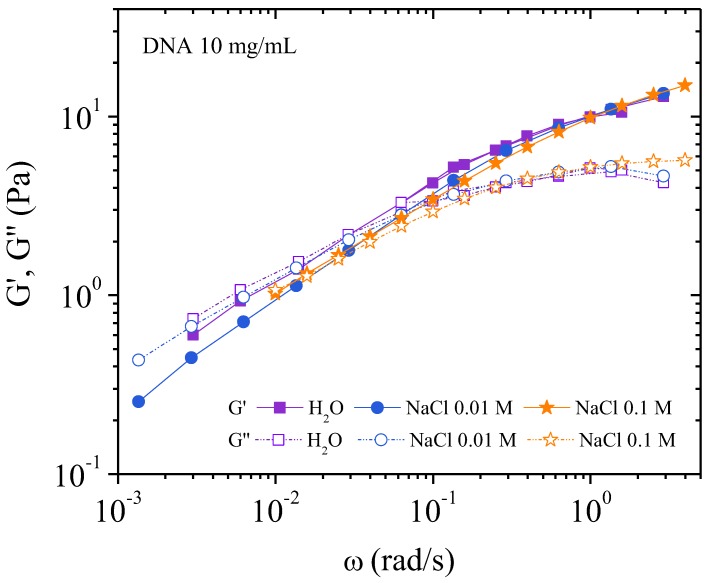
Elastic and viscous moduli obtained by dynamic oscillatory rheology for 10 mg/mL DNA solution at different ionic concentrations: 0, 0.01, and 0.1 M NaCl. (*T* = 25 °C).

At low frequencies the behavior, as observed by dynamic rheological measurements, is nearly identical in the three solvents. An original behavior is observed in Figure 10. 

*G*’ and *G*’’ are superimposed in water and additionally, *G*’ = *G*’’ up to *ω*~0.08 rad/s. Below *ω*~0.08 rad/s, *G*’ (*ω*) = *G*’’ (*ω*) = *G*^0.5^ the exponent equals 0.5, in agreement with the criterion of Winter et al. [21,22,23]. The criterion of Winter et al. states that a network can form, for low frequencies, in the semidilute entangled DNA solution. This network is caused by strong electrostatic repulsion impeding the movement of single chains until some critical stress is applied. In 0.01 and 0.1 M NaCl, a slight decrease of *G*’ is observed indicating the decrease of the electrostatic repulsion. At higher frequency, *G*’ > *G*’’ is related to the modification of the supramolecular structure of the fluids corresponding to transition from solid like behavior to a liquid flowing phase which occurs above the critical gel condition (*ω* = 0.08 rad/s). Above 0.1 rad/s, the moduli overlap in water and NaCl solutions as described by Winter and Mours for a critical gel (Figure 12 of a previous paper [22]). In the range of high frequencies *G*’’ is nearly constant without influence of salt content being unaffected by the degree of cross-linking, indicating the behavior of a classical viscoelastic fluid.

##### Flow Measurements

**Figure 11:**
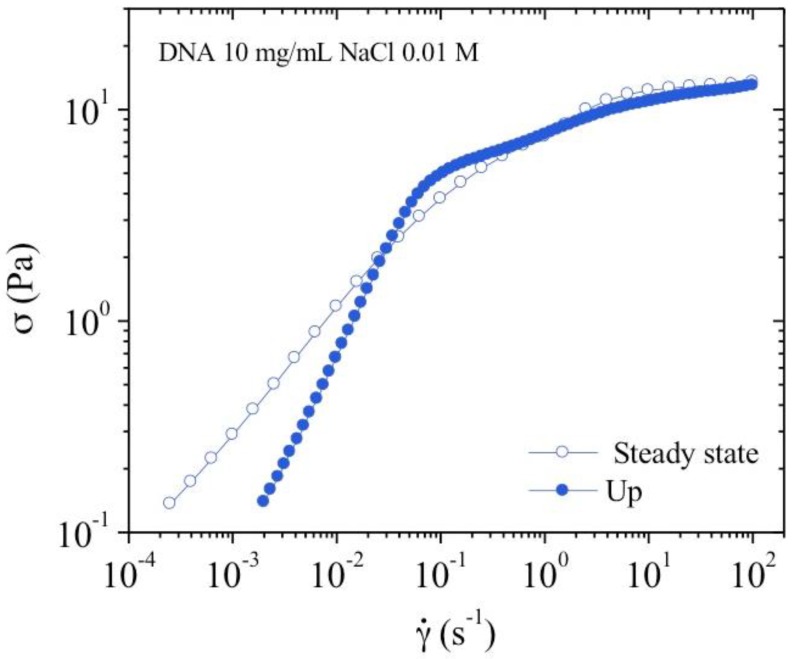
Shear stress as a function of the shear rate showing thixotropy and apparent yield stress of 10 mg/mL DNA solution in 0.01 M NaCl at *T* = 25 °C. Filled symbols where obtained by applying a rapid shear rate increase from 0 to 100 s^−1^ within 160 s, directly followed by a decreasing ramp within a time of 3000 s (open symbols).

It is difficult to reproduce viscosity measurements at low shear rate for concentrated systems [24,25,26,27,28]. This is illustrated in Figure 11 in 0.01 M NaCl. Firstly, an increasing shear rate is imposed resulting in a stress versus shear rate dependence with a slope of 1 up to around 0.1 s^−1^, corresponding to an apparent Newtonian plateau. Above 0.1 s^−1^, shear stress follows a smooth evolution up to 100 s^−1^ corresponding to a bulk flow regime. On the decreasing shear rate ramp, the shear stress decreases taking values larger than those obtained during the increasing ramp at low shear rate. The increase behavior is characteristic of slipping at the walls and thixotropy occurring in more concentrated solutions [24,29]. The viscosity of the sample is dependent on its shear history, therefore obtaining a reproducible and significant value is difficult. These difficulties are illustrated in Figure 11. In concentrated system, those generic flow curves are the signature of an apparent yield stress fluid with slipping as confirmed hereinafter. In fact, the yield stress is proportional to the elastic shear modulus [24], which is nearly the same in the three solvents with a small influence of electrostatic interactions due to the presence of external salt (Figure 10). 

**Figure 12:**
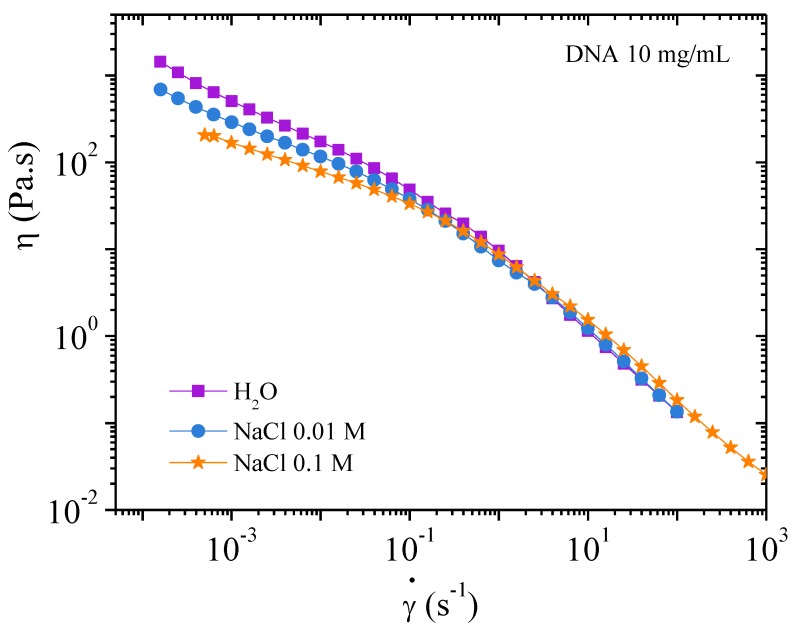
Flow viscosities as a function of the shear rate for DNA 10 mg/mL solutions in the three solvents: H_2_O, 0.01, and 0.1 M NaCl. Values are taken on the decreasing shear rate ramp.

**Figure 13:**
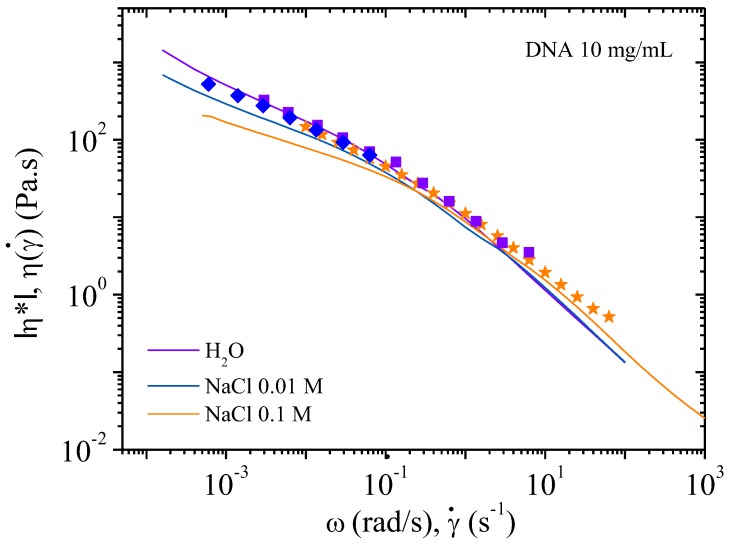
Cox–Merz overlay for 10 mg/mL DNA solution at 25 °C in different ionic concentrations: 0, 0.01, and 0.1 M NaCl. Continuous lines represent the flow viscosities and the dots represent dynamic oscillatory viscosities measurements.

Figure 12 is a plot of viscosity against shear rate. The points were obtained while decreasing the shear rate. It can be seen that the values are lower for solutions with added salt: this is expected behavior for polyelectrolytes. In all three cases, viscosity increases at low shear rate in agreement with the existence of a yield stress. Over 10^−1^ s^−1^ the viscosities in the three solvents are overlapping in agreement with the behavior in dynamic rheological measurements. The flow viscosity and complex viscosity from the oscillatory behavior are shown in Figure 13. 

From these data, the complex viscosity fairly agrees with the shear viscosity measurements following the Cox–Merz rule (Figure 10 and Figure 12) for which *G*’ and *G*’’ overlap over *ω* = 10^−1^ rad/s. At low frequency, in the range of gel-like behavior, the Cox–Merz rule is no more valid in the presence of external salt probably due to slip at the wall in absence of any supramolecular structural change as indicated in Table 2. 

**Figure 14:**
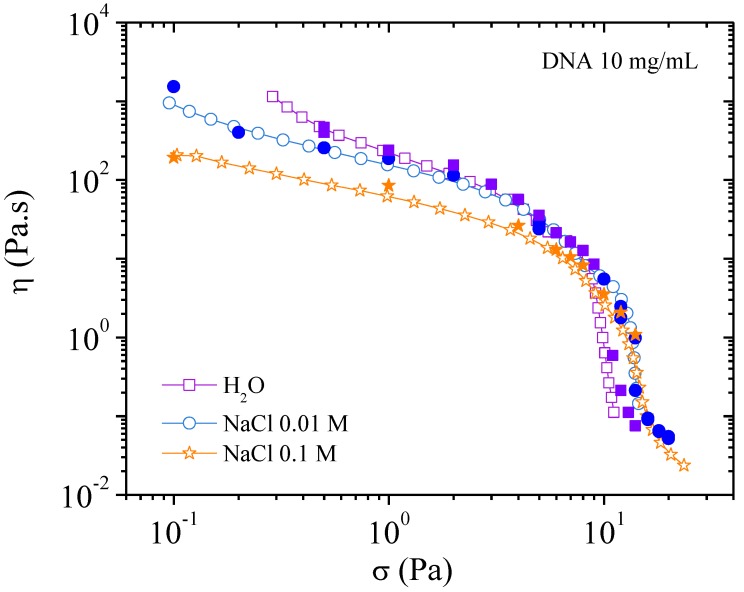
Viscosity as a function of the shear stress at 25 °C in H_2_O, 0.01, and 0.1 M NaCl. Measures were performed on decreasing shear rate sweep (open symbols) and in the stationary state from creep step (filled symbols).

**Figure 15:**
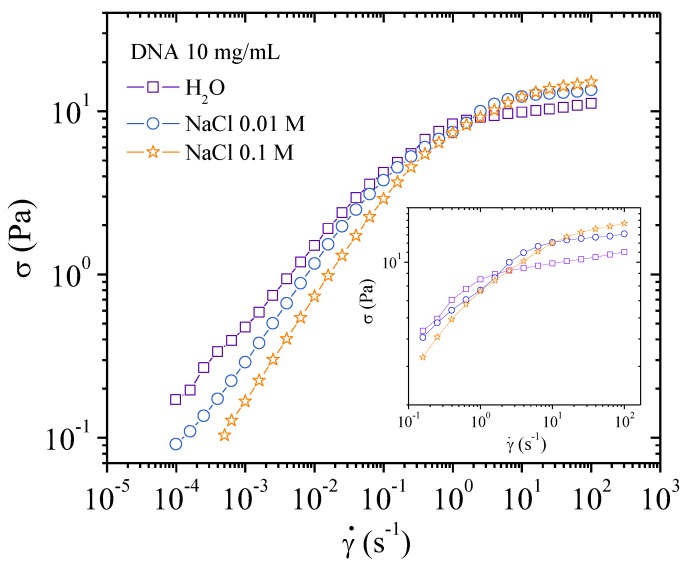
Shear stress as a function of the shear rate for DNA 10 mg/mL solution at 25 °C in H_2_O, 0.01, and 0.1 M NaCl.

**Figure 16:**
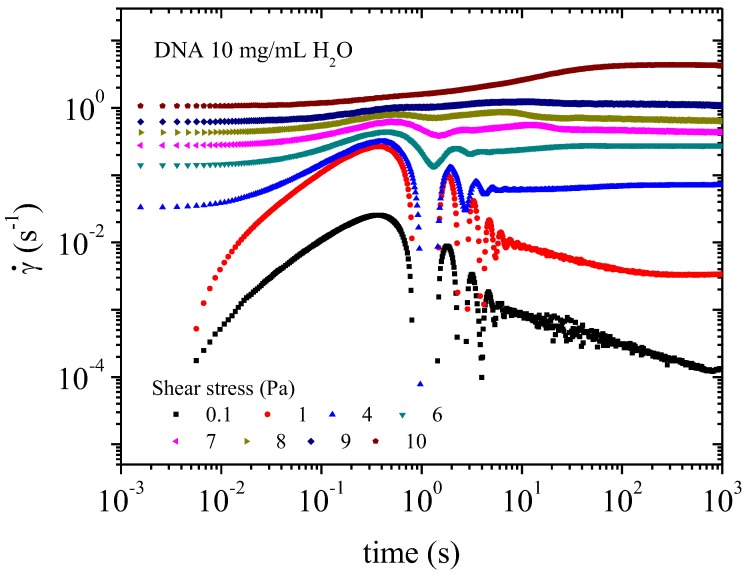
Transient behavior for DNA solution 10 mg/mL in water: creep experiment giving the shear rate as a function of time for each stress applied from 0.1 to 10 Pa.

**Figure 17:**
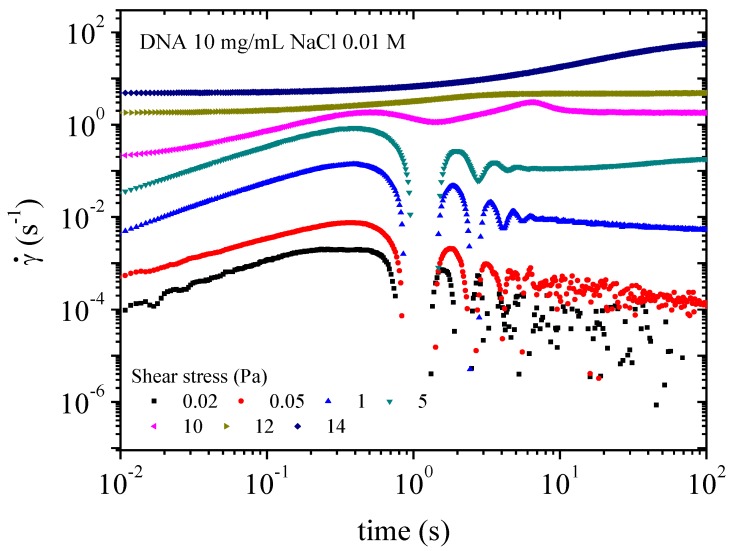
Transient behavior for DNA solution 10 mg/mL in water: creep experiment giving the shear rate as a function of time for each stress applied from 0.1 to 10 Pa.

**Figure 18:**
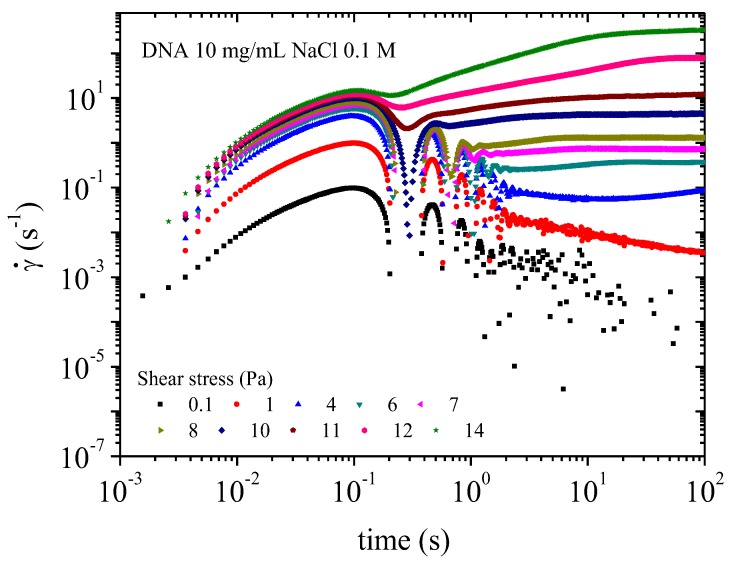
Transient behavior for DNA solution (10 mg/mL) in 0.1 M NaCl: creep experiment giving the shear rate as a function of time for each stress applied from 0.1 to 14 Pa.

To relate the rheological behavior with supramolecular organization, viscosity is plotted as a function of the applied stress in Figure 14. It is shown that the stress needed to orientate the electrostatic network with flow direction in water is around 10 Pa much larger than for 4 mg/mL DNA solution (~2 Pa, Figure 9). In these curves, two series of values are plotted and are found in good agreement: values from flow experiments on the decreasing step and values from the stationary state from creep step (see later in Figure 16, Figure 17 and Figure 18). In the presence of 0.01 M NaCl, the orientation stress increases up to almost 15 Pa. A higher energy is required to align the system due to partial screening of the electrostatic interactions (Figure 14). This is in agreement with the lower order parameter as shown in X-rays and the appearance of a plateau in the *σ*($\overset{´}{\gamma }$) representation (Figure 15). In the presence of 0.1 M NaCl, a smooth variation of the viscosity as a function of stress is obtained and is in good agreement with absence of a plateau in *σ*($\overset{´}{\gamma }$) representation (Figure 15).

In the representation *σ*($\overset{´}{\gamma }$), the presence of a plateau corresponds to the appearance of textures as shown in Figure 5a,b. In the same conditions as in rheological experiments, in water, texture occurs directly over 1 s^−1^ (i.e., 5 Pa, Figure 5a) and continues forming at a nearly constant shear stress (10 Pa) at which the viscosity drops (Figure 14) giving a plateau in the *σ*($\overset{´}{\gamma }$) curve (Figure 15). In a 0.01 M NaCl DNA solution a transition occurs ~5 Pa. This corresponds to the appearance of an equivalent texture (Figure 5b). The transition is completed at a constant shear stress (12 Pa) before the viscosity drops. This drop in viscosity corresponds to a plateau in the *σ*($\overset{´}{\gamma }$) curve. The vertical viscosity drop is longer in water than in 0.01 M NaCl: this is due to the decrease of electrostatic interchain interactions in the presence of external salt. In 0.1 MNaCl DNA solution, the viscosity decreases progressively as a function of stress applied (Figure 14). Unlike in water and 0.01 M NaCl solutions, no texture is observed (Figure 5c). Above shear stress of 8 Pa, a heterogeneous birefringent solution organization across the entire volume is obtained indicating the orientation of large associated domains going to fully birefringent solution at high shear stress. Irrespective of the solvent, for shear stresses greater than 15 to 20 Pa, complete orientation of DNA forms a pseudonematic phase as suggested (birefringence is maximum) [11].

#### 3.3.3. Transient Regime

The transitory state is defined when constant shear stress is applied. Creep experiments enable this regime to be studied. In water, at 10^−2^ Pa, no flow occurs (Figure 16). After transient inertia viscoelastic coupling, the shear rate decreases until 10^−4^s^−1^ for 10^3^ s, indicating a solid like behavior in water (as indicated in Figure 10). The sample starts flowing at 10^−1^ Pa corresponding to 3 × 10^−3^ s^−1^. Flow is accompanied by a change in the polymer structure when the network is ruptured as evidenced by a rapid decrease in viscosity (Figure 12). The first oscillations in Figure 16 occur at a characteristic time, which is progressively shifted for stress applied higher than 5 Pa. At 7 Pa the oscillations disappear giving a bump around 10 s corresponding to the transition in shear stress (Figure 14). Over 9 Pa, corresponding to the pseudo-plateau in shear stress versus shear rate representation, when the texture is observed the supramolecular structure is stabilized very rapidly.

A similar behavior is observed in the presence of 0.01 M NaCl. At very low stress (circa 0.02 Pa) the sample behaves as a solid (Figure 17). The sample begins to flow at a lower yield stress than the sample in water and is faster to reach the stationary state. For samples in 0.01 M NaCl solution flow occurs at circa 1 Pa, and the stationary state is obtained after a maximum of 100 s. When stress above 10 Pa is applied the sample exhibits a textured structure under birefringence. Physically this behavior is caused by a looser electrostatic network providing less hindrance to movement than for DNA in water solution corresponding to lower viscosity (Figure 12). 

In 0.1 M NaCl, the flow occurs at very low shear stress corresponding to an apparent yield stress lower than 0.1 Pa (Figure 18). In this salt concentration, the supramolecular organization changes to a looser structure. 

Irrespective of solvent conditions, the DNA solutions at 10 mg/mL present a yield stress at very low shear stress in agreement with dynamical and flow measurements (Figure 10 and Figure 12).

## 4. Conclusions

The influence of the external salt concentration on the rheology and supramolecular structure of DNA solutions was discussed in this paper. Rheological measurements were performed with SAXS and birefringence experiments. In water, strong interchain electrostatic repulsions induced the existence of a correlation peak between chains in solution. This correlation peak is progressively depressed in the presence of external salt. Two DNA concentrations in the semi-entangled regime were chosen: 4 mg/mL, which exhibits birefringence at high shear rates, but no particular texture is observed; and 10 mg/mL, where clear supramolecular structure was evidenced previously in 0.01 M buffer solution [11].

At 4 mg/mL in water, the *G*’ modulus and the viscosity under flow are higher than in presence of salt. Viscosity decreases steeply around a stress of 5 Pa and birefringence occurs over 50 s^−1^ corresponding to alignment of all the chains in the flow. Salt in solution changes the rheological behavior due to screening of electrostatic repulsions. The local organization is depressed but it is shown that the dynamic modulus and flow viscosity are lower in 0.01 M NaCl than in 0.1 M NaCl. In the case of 0.1 M NaCl concentration, long-range electrostatic interactions are absent and steric interactions dominate in the entangled semi-rigid chain assembly, inducing higher *G*’ and viscosity in the Newtonian plateau.

Irrespective of ionic concentration, increasing the stress progressively allows orienting the interacting chains and the curves obtained for the different solvents superimpose over a stress around 2 Pa (Figure 9). Larger stresses correspond to the entrance in the birefringent regime where all the chains orient in the flow over 4 Pa. Clearly, from Figure 9, a smooth transition is obtained confirming the absence of plateau on *σ*($\dot{\gamma }$) representation.

At 10 mg/mL, an electrostatic network with *G*’ (*ω*) = *G*” (*ω*) is demonstrated from dynamic rheology at frequency lower than 0.08 rad/s in water; electrostatic interactions decrease progressively in presence of external salt being progressively substituted by steric interactions. Then, an apparent yield stress is found from stress measurement at low shear rate and confirmed in transient experiments in water and to a lower degree in NaCl. Due to this mechanism, viscosity measurements as a function of the shear rate are delicate and the values must be taken on the decreasing step in shear rate. Due to this gelation mechanism, observation time dependence involved in experiments under stress implies change in the supramolecular structure and dynamic of the systems.

The curves giving the viscosity as a function of the shear stress applied presents a two steps transition: (i) the first at approximately 4 Pa (over 1 s^−1^) where texture starts; the second at 10 Pa in water; (ii) at 4 Pa and approximately 12 Pa in 0.01 M NaCl, respectively, corresponding to texture appearance with large decrease in viscosity at constant shear stress. The formation of the texture corresponds to a plateau in the shear stress (shear rate) representation. Textures in 0.01 M NaCl are less visible than in water corresponding to a lower organization of stiff chains. Nevertheless, in 0.1 M NaCl, smooth variation of the viscosity with shear stress with looser organization (no texture as shown in water and 0.01 M NaCl) is obtained with no plateau in the stress versus shear rate representation. In 0.1 M NaCl, heterogeneous birefringent solution formed by large domains orientated in the flow. Whatever the ionic conditions, at higher shear stress, all the chains orient in the flow with a low viscosity and the solutions are birefringent corresponding to a pseudonematic supramolecular organization of the system. 

This work clearly describes the influence of polymer concentration and external ionic concentration on the rheological behavior and supramolecular structure of this original stiff DNA polymer. It is demonstrated that at higher polymer concentration, a plateau is obtained in the *σ*($\overset{´}{\gamma }$) representation as soon as long-range electrostatic interactions and texture exist. An important point is that the rheology is dependent on the history of the samples at higher polymer concentration (as seen in the example of DNA solution at 10 mg/mL). The experimental conditions need to be precisely controlled, especially the ramp speed in viscosity and shear rate experiments, to obtain robust data. 

For the first time, the presence of an electrostatic network is demonstrated, especially in water at 10 mg/mL DNA concentration where electrostatic repulsions are at a maximum. In addition, for all samples studied, it is shown that the rheological behavior is particularly original at low flow shear rate and low dynamic frequency applied.

## Figures and Tables

**Table 1:** Estimation of the Debye screening length *κ*^−1^ expressed in nm.

Polymer Concentration (mg/mL)	*κ*^−1^_water_ (nm)	*κ*^−1^_0.01 M NaCl_ (nm)	*κ*^−1^_0.1 M NaCl_ (nm)
4	8.42	2.83	0.94
10	5.36	2.61	0.93

**Table 2:** Influence of polymer and ionic concentration on the critical shear rate for birefringence and texture formation.

Solvent Properties	H_2_O	0.01 M NaCl	0.1 M NaCl
4 mg/mL Birefringence	*σ* = 5 Pa	*σ* = 4.6 Pa	*σ* = 4.5 Pa
4 mg/mL Texture	NO	NO	NO
10 mg/mL Birefringence	$\overset{´}{\gamma }$ > 1 s^−1^ *σ* = 8 Pa	$\overset{´}{\gamma }$ > 1 s^−1^ *σ* = 9 Pa	$\overset{´}{\gamma }$~100 s^−1^ *σ* = 10 Pa
10 mg/mL Texture	$\overset{´}{\gamma }$ ~1–10 s^−1^ *σ* = 10 Pa	$\overset{´}{\gamma }$ ~10 s^−1^ *σ* = 15 Pa	Heterogeneous birefringent domains *σ* = 8 Pa

**Table 3:** Rheological parameters for different ionic concentrations.

Characteristic Parameters	H_2_O	0.01 M NaCl	0.1 M NaCl
*G*_0_ (Pa)	0.84	0.68	0.78
*ω*_0_ (rad/s)	0.31	0.82	0.59
*η*_0_ (Pa.s) for $\dot{\gamma }\to 0\;s^{-1}$	18 at $\overset{´}{\gamma }$ = 10^−3^s^−1^	4.1	7.2

## Supplementary Materials

The following are available online at http://www.mdpi.com/2073-4360/10/11/1204/s1, Video S1, S2: DNA solutions 10 mg/mL in water and in 0.1 M NaCl under shear are joined in SI.

## Abbreviations

The following abbreviations are used in this manuscript:

DNA	Deoxyribonucleic Acid
SAXS	Small-angle X-ray scattering
