# Supplementary material for: Role of Electrostatic Interactions on Supramolecular Organization in Calf-Thymus DNA Solutions under Flow
Source: Polymers (Basel). 2018 Oct 28;10(11):1204. doi: 10.3390/polym10111204 (PMC6290628; doi:10.3390/polym10111204)
Supplement: Supplementary file 1 [file polymers-10-01204-s001.pdf]

Article

Role of electrostatic interactions on supramolecular organization in calf-thymus DNA solutions under flow

L. Mónica Bravo-Anaya[1,2], Denis C. D. Roux[1], J. Félix Armando Soltero Martínez[2], Francisco Carvajal Ramos[3], Frédéric Pignon[1], Oonagh Mannix[4], Marguerite Rinaudo*[5]

Received: date; Accepted: date; Published: date
Academic Editor: name

[1] University Grenoble Alpes, CNRS, Grenoble INP, LRP, F-38000 Grenoble (France); monik_ayanami@hotmail.com, Denis.Roux@univ-grenoble-alpes.fr, frederic.pignon@univ-grenoble-alpes.fr
[2] Universidad de Guadalajara, Departamento de Ingeniería Química. Blvd. M. García Barragán #1451, C.P. 44430, Guadalajara, Jalisco (México); jfasm@hotmail.com
[3] Universidad de Guadalajara, CUTonalá, Departamento de Ingenierías, Nuevo Periférico #555 Ejido San José Tatepozco, C.P. 45425, Tonalá, Jalisco (México); iq_fcr@yahoo.com.mx
[4] European Synchrotron Radiation Facility, 38000 Grenoble (France); oonmannix@gmail.com
[5] Biomaterials applications, 6 rue Lesdiguières, 38000 Grenoble (France); marguerite.rinaudo@sfr.fr

From figure 5: two videos for solutions in water and in 0.1M NaCl are available to download using the following dropbox links:

DNA 10 mg/mL H_2O

https://www.dropbox.com/s/f4h4p2zeul0svl3/ADN10gperLH2O.avi?dl=0

DNA 10 mg/mL 0.1 M NaCl

https://www.dropbox.com/s/z7gz1cf5baxpjr8/ADN10gpL01MNaCl.avi?dl=0
